# Implications of “Too Good to Be True” for Replication, Theoretical Claims, and Experimental Design: An Example Using Prominent Studies of Racial Bias

**DOI:** 10.3389/fpsyg.2016.01382

**Published:** 2016-09-22

**Authors:** Gregory Francis

**Affiliations:** Department of Psychological Sciences, Purdue UniversityWest Lafayette, IN, USA

**Keywords:** racial bias, publication bias, statistics, questionable research practices, replication

## Abstract

In response to concerns about the validity of empirical findings in psychology, some scientists use replication studies as a way to validate good science and to identify poor science. Such efforts are resource intensive and are sometimes controversial (with accusations of researcher incompetence) when a replication fails to show a previous result. An alternative approach is to examine the statistical properties of the reported literature to identify some cases of poor science. This review discusses some details of this process for prominent findings about racial bias, where a set of studies seems “too good to be true.” This kind of analysis is based on the original studies, so it avoids criticism from the original authors about the validity of replication studies. The analysis is also much easier to perform than a new empirical study. A variation of the analysis can also be used to explore whether it makes sense to run a replication study. As demonstrated here, there are situations where the existing data suggest that a direct replication of a set of studies is not worth the effort. Such a conclusion should motivate scientists to generate alternative experimental designs that better test theoretical ideas.

## Introduction

The last 5 years have been brutal for psychology. A high profile case of fraud (Stapel and Lindenberg, 2011) led to claims that the field should have recognized many deficiencies in the reported findings (Levelt et al., 2012). At around the same time, one of the top journals in the field published a series of experiments purportedly showing evidence of precognition: that people could acquire information from the future and use it in the present (Bem, 2011). While this case did not seem to be due to fraud, it became a poster child for a set of improper methods in scientific investigations that sometimes go by the term p-hacking (Simmons et al., 2011) or **questionable research practices** (QRPs, John et al., 2012). These methods violate the rules of data collection, reporting, or statistical analysis, and thereby potentially undermine the conclusions derived from the reported empirical data.

KEY CONCEPT 1. Questionable research practices: Researchers often have an opportunity to adjust the sampling procedure, modify the data analysis, selectively report some findings, or adjust their theoretical ideas after observing the data. This kind of flexibility sometimes undermines the validity of the conclusions derived from the data.

Some concerned scientists have taken an empirical approach to the perceived crisis in psychology. A series of new experiments have attempted to repeat well-known empirical studies to judge whether the reported effects are robust. There have been notable successes (e.g., Röer et al., 2013) but equally notable failures to replicate important findings (Doyen et al., 2012; Galak et al., 2012; Shanks et al., 2013; Johnson et al., 2014; Ranehill et al., 2015). Even when replications find evidence for an effect, it is often determined to be much smaller than the original report (e.g., Alogna et al., 2014). In one large-scale effort, the Open Science Collaboration (2015) attempted to replicate 97 prominent experimental outcomes, but was only able to meet the traditional criterion for **statistical significance** in 25% of the social psychology experiments (cognitive psychology did better with a still disappointing 50% success rate).

KEY CONCEPT 2. Statistical significance: A somewhat arbitrary conclusion about the existence of an effect (e.g., a difference in population means) based on observing an empirical result (e.g., a difference of sample means) that would be rather unusual if there were no effect.

There is undeniable value in these empirical investigations, but they are expensive in terms of time and effort (for both scientists and participants). Moreover, proponents of the original findings often complain that the replicators messed up the experiment (Schnall, 2014); and this criticism is sometimes levied even when the proponents approved the **replication** design (Baumeister and Vohs, 2016). Such charges can be difficult to refute because the original studies often do not fully describe the necessary conditions for producing an effect. Because of these issues, using empirical investigations to sort out the literature is a daunting task. Indeed, even the large scale Open Science Collaboration (2015) only replicated a single experiment from papers that often had multiple studies.

KEY CONCEPT 3. Replication: An effort to reproduce experimental conditions, measurements, and analyses to further explore or verify a previous study. Success is usually determined according to whether the new experiment generates results with the same general properties as the original study. When experimental outcomes are based on statistics, success is necessarily a probabilistic property.

As part of the perceived crisis, scientists have gained a better understanding about how various types of biases can undermine their theoretical claims. Unfortunately, with this knowledge the story about some past research seems to only get worse. Lane et al. (2016) reported that when they analyzed both their published and (previously) unpublished studies on the effects of oxytocin the main effect appeared to be non-existent. Using new types of meta-analyses (Stanley and Doucouliagos, 2014) that take into account the influence of publication bias, Carter and McCullough (Carter and McCullough, 2014) found no support for the “ego-depletion effect” (the idea that one has a limited resource for self-control). A large-scale, pre-registered, multi-site replication investigation concluded that if the ego-depletion effect exists, then it is extremely tiny (Hagger et al., 2015). As a center-piece of many theories of social cognition, this negative finding led Inzlicht (2016), who has based some of his work on the theory, to conclude, “I feel like the ground is moving from underneath me, and I no longer know what is real and what is not.” He continued, “During my dark moments, I feel like social psychology needs a redo, a fresh start. Where to begin, though?”

A purely experimental approach does not seem like a reasonable way to begin a fresh start to the field because setting up replications for an entire field would be a daunting task and would take up so many resources as to essentially halt any other kind of research. A more practical beginning is to judge the quality of past findings by looking for inconsistencies in a set of findings; but identifying these inconsistencies requires a nuanced understanding about the relation between experimental results and theoretical conclusions. The conclusions about findings from an empirical study often depend on statistical significance as a criterion for “success.” From this perspective, a failure to show significance is cause for concern and casts doubt on the existence of an effect. This concern is valid, but one also has to consider that some empirical failures are expected because every study has a random component (typically in selecting a sample of participants). Because experimental failures are expected, their absence can indicate problems in reporting, sampling, or analysis of a set of empirical findings. For example, Francis (2012a) and Schimmack (2012) used statistical analyses to show that Bem's precognition studies seemed “too good to be true.” This kind of analysis, called the **Test for Excess Success** (TES), uses the reported data to estimate the probability of success for replication experiments with the same design and sample size(s). If this success probability is low, then scientists should be skeptical about the validity of the original findings. Systematic investigations with the TES analysis revealed that over 80% of articles with four or more experiments in two high-profile journals had problems similar to Bem's precognition studies (Francis, 2014a; Francis et al., 2014). For a single article, a TES analysis can often be completed in an afternoon, which is several orders of magnitude faster and easier than running empirical replications of a set of studies.

KEY CONCEPT 4. Test for excess success: A statistical analysis that uses findings from a set of experiments to estimate the probability that a direct replication would reproduce the same pattern of successful outcomes. When the probability is low, the analysis raises doubts about the validity of the original experiment set.

There may be little motivation to perform a TES analysis for findings with little impact. Scientists get the most benefit by doing a TES analysis (or an empirical replication study) that examines findings and theories that have influenced the field either by motivating research on important topics or by encouraging applications. Francis (2015) presented one such TES analysis that looked at several prominent papers (Eberhardt et al., 2004; Goff et al., 2008; Williams and Eberhardt, 2008) that investigated properties of racial bias on perception. The findings and claims in these papers have been widely publicized as having important implications for understanding racial bias (MacArthur Foundation, 2014; Dreifus, 2015; Noë, 2015), and they have been part of training programs used by police departments to better understand and mitigate racial bias (Laszlo and Fridell, 2012).

Rather than simply repeat the TES analysis in Francis (2015), here I want to look in detail at one of the experiments and discuss the relationship between the empirical findings and the theoretical conclusions. A similar kind of analysis indicates that, based on the information in the original experiments, replications of the studies in Eberhardt et al. (2004) are unlikely to be fruitful even if the sample sizes are dramatically increased.

## Estimating an experiment's success probability

An important aspect in determining success probability is the definition of “success.” In many scientific reports success for empirical experiments is relative to the final theoretical claims that are based on those experimental results. Thus, to create a TES analysis for an experiment one must identify the theoretical claims of the experiment. Fortunately, in many cases, the original manuscript is clear about the theoretical claims; indeed, identification of those claims is often the main point of a scientific article.

In study 1 from Eberhardt et al. (2004), participants were exposed to one of three subliminal priming conditions: white face priming, black face priming, or no face priming. After the prime exposure, participants were asked to identify outline shapes that were embedded in visual noise. The noise was gradually reduced with successive image frames, and the dependent variable was the number of frames presented before the participant reported that they could see the shape. Across trials, the object was sometimes crime-relevant (e.g., a gun or a knife) and other times crime-irrelevant (e.g., a camera or a book). Based on their analysis of the data, Eberhardt et al. (2004) concluded, “both Black and White primes tune the detection of crime-relevant objects, yet in opposite directions” (p. 881).

**Figure 1:**
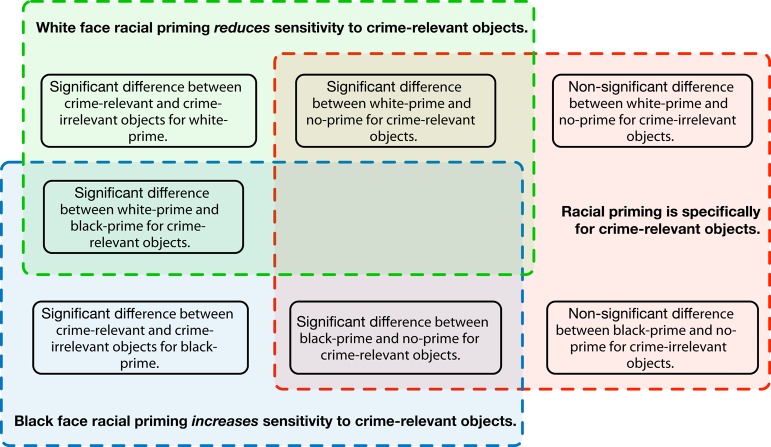
**The three theoretical claims and the seven hypothesis tests used to support those claims in study 1 of Eberhardt et al. (2004)**.

Figure 1 breaks down the theoretical claims of Eberhardt et al. (2004) into three statements and identifies the seven statistical tests they used to support those claims.

1. Black face racial priming *increases* sensitivity to crime-relevant objects. This claim is based on three statistically significant hypothesis tests. One test compared the number of frames for detection of crime-relevant objects for white-prime and black-prime participants. A second test compared the number of frames for detection of crime-relevant objects for black-prime and no-prime participants. A third (within-subjects) test compared the number of frames for detection of black-prime participants for crime-relevant objects against crime-irrelevant objects. Eberhardt et al. (2004) described this particular claim as a prediction based on their hypothesis that stereotypic associations influence visual processing.
2. White face racial priming *decreases* sensitivity to crime-relevant objects. This claim is based on three statistically significant hypothesis tests. One test compared the number of frames for detection of crime-relevant objects for white-prime and black-prime participants. A second test compared the number of frames for detection of crime-relevant objects for white-prime and no-prime participants. A third (within-subjects) test compared the number of frames for white-prime participants for detection of crime-relevant objects against crime-irrelevant objects. Eberhardt et al. (2004) did not report that their hypothesis predicted this relationship, but it nevertheless is a (narrow) theoretical claim about stereotypic associations and visual processing in this kind of study. In particular, although other studies did not report finding a similar relationship for white priming, those different outcomes were attributed to methodological differences in stimuli and tasks rather than being due to noise from random sampling, and this interpretation forms part of the theoretical claims in Eberhardt et al. (2004) about this experiment.
3. Racial priming is specifically for crime-relevant objects. This claim is based on two significant and two non-significant hypothesis tests. The significant tests were for differences between the black-prime and no-prime conditions and between the white-prime and no-prime conditions for the crime-relevant objects. The non-significant tests compared differences between the black-prime and no-prime conditions and between the white-prime and no-prime conditions for crime-irrelevant objects. Although it is somewhat unusual to base theoretical conclusions on null results, Eberhardt et al. (2004) are specific about the role of these tests, “As predicted, there was no significant effect of race prime on crime-irrelevant objects” (p. 880).

So, success for study 1 of Eberhardt et al. (2004) required five significant outcomes and two non-significant outcomes. If any of these outcomes were unsuccessful, it would call in to doubt at least some of the theoretical claims made by Eberhardt et al. (2004). Of course, just due to random sampling, one would not always expect all of these outcomes to be successful. A TES analysis estimates the probability that an experiment like this one would produce full success (significance or non-significance, as appropriate) across all the tests.

**Figure 2:**
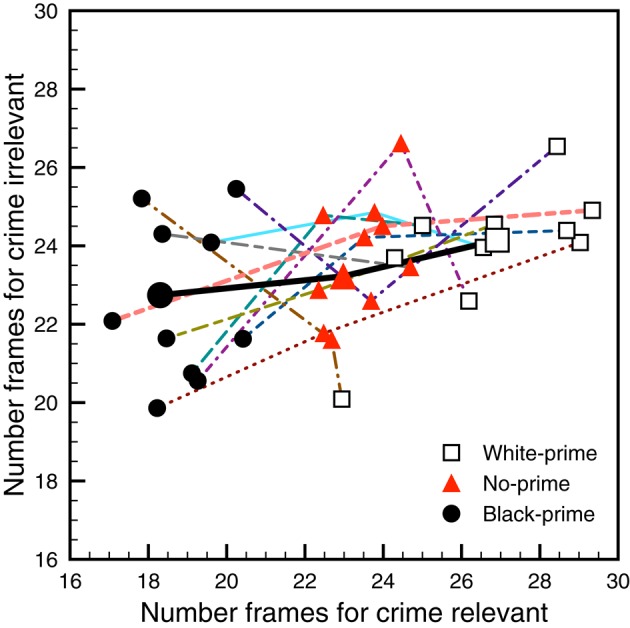
**Ten simulated experimental findings for study 1 of Eberhardt et al. (2004)**. The x- and y-axes correspond to the sample mean value for the crime-relevant and crime-irrelevant conditions, respectively. The different symbols correspond to the different priming conditions, as indicated in the legend. A line connects points from the same simulated experiment. The solid black line with large symbols corresponds to the findings reported by Eberhardt et al. (2004).

An estimate of the success probability of all the outcomes in study 1 of Eberhardt et al. (2004) was computed by simulated experiments that use the reported sample means, standard deviations, and correlations (of within-subject measures) as representative of population values. In the simulations 100,000 experiment samples were drawn from normal distributions with the same sample sizes (13, 12, and 14 subjects for the white-prime, no-prime, and black-prime conditions, respectively) as study 1 of Eberhardt et al. (2004); and these samples were then subjected to the tests identified in Figure 1. Each of the 10 dashed lines in Figure 2 plots the means for the various conditions that were generated by one simulated experiment. The black solid line with large symbols corresponds to the means reported by Eberhardt et al. (2004). As is to be expected with such small sample sizes, the simulated means vary quite a bit around the originally reported means. Moreover, there is so much variability that oftentimes the significance status “flips” for one or more of the tests. Only one simulated experiment in Figure 2 had full success; it corresponds to the peach-colored dashed line. Out of the 100,000 simulated experiments, only 16,294 produced success for all seven statistical tests identified in Figure 1. Thus, if the population means and standard deviations are similar to those reported by Eberhardt et al. (2004), then experiments like these have an estimated success rate of around 0.163. R source code for these simulations is available in the Supplemental Material.

This low estimated success rate indicates that, based on the available data, it is rather unlikely that a replication study with the same sample sizes will produce the pattern of results that Eberhardt et al. (2004) used to support their theoretical claims.

## Estimating success for a set of experiments

The general conclusions in Eberhardt et al. (2004) were based on not just the outcomes from study 1, but also from an additional four experiments. As Eberhardt et al. (2004) noted at the beginning of their general discussion, “Across five studies, we have shown that bidirectional associations between social groups and concepts can guide how people process stimuli in their visual environment” (p. 889).

The same kind of success rate estimation process can be applied to the other four experiments in Eberhardt et al. (2004), and it finds estimated success rates of 0.380, 0.575, 0.450, and 0.212 for experiments 2–5, respectively. Only study 3 has a better than 50% chance of a replication study being fully successful; and study 5 has only around a 20% chance of producing results that fully agree with the outcomes described in Eberhardt et al. (2004). Details of the analyses and R source code are in Francis (2015).

This analysis suggests that none of these experiments were well crafted to provide empirical support for the theoretical ideas described in Eberhardt et al. (2004). Given the expected low success rates across all the experiments, it is rather surprising that every experiment was fully successful. Indeed, since the experiments were derived from independent samples, the probability of full success across all five studies is the product of the success probabilities, 0.003, which indicates that full success across all five experiments should be a very uncommon outcome.

In their general discussion, Eberhardt et al. (2004) noted that, “We found remarkably consistent support for both visual tuning and bidirectionality using three different paradigms that incorporated three different types of participant judgments as well as both image and word stimuli, both student and police officer participant populations, both positive and negative concepts, and both explicit and implicit measures” (p. 889). The TES analysis suggests that the support is not just “remarkably consistent” but “shockingly consistent,” and perhaps even “unbelievably consistent.” Given the variability that should be present simply due to random sampling, the uniform success across all five experiments reported by Eberhardt et al. (2004) is rather unbelievable. To the astute reader it suggests a problem with the sampling procedures, a problem with the analyses, or a problem of not reporting the unsuccessful studies or outcomes that almost surely occurred. Regardless of the cause, readers should be skeptical about the theoretical claims that are based on this set of experiments.

Francis (2015) performed similar calculations for two other papers that addressed similar topics (Goff et al., 2008; Williams and Eberhardt, 2008) and found similar estimates of low success rates: 0.048 and 0.07, respectively. Readers are advised to be skeptical of the theoretical claims that were based on the reported findings in those studies.

## What happened in these studies?

It is important to recognize the specific criticism derived from the TES analysis: it is that the empirical findings seem too successful to plausibly support the theoretical claims. Excluding the possibility of a chance occurrence, one can interpret the seeming excess success by supposing that there are flaws in the empirical findings (sampling or reporting problems) or flaws in the development of the theoretical claims (HARKing or model over fitting to data). In either case, a replication study that used proper sampling and reporting to test the theoretical claims with the same tests and sample sizes is unlikely to support the full set of claims.

The problems with the findings in Eberhardt et al. (2004) seem representative of problems across the broad field of social psychology. As noted in the introduction, scientists often seem to use a variety of QRPs to convince themselves that their data support their theoretical ideas. The TES analysis can identify the presence of these approaches, but cannot identify what kind of QRPs were used for a particular data set. It is important to note that use of a QRP is not fraud. As Gelman and Loken (2014) noted, it is possible for scientists to introduce QRPs without realizing it.

Indeed, some standard approaches to science seem to encourage some types of QRPs. For example, a common scientific attitude is to “follow the data” when generating theoretical claims, but this is actually poor advice in many situations. Consider a hypothetical outcome for the findings in study 1 of Eberhardt et al. (2004) where (contrary to what was actually reported) for crime-relevant objects the participants in the white-prime condition do not show a priming effect that differs from the participants in the no-prime condition. A scientist observing such a pattern might conclude that although black-priming increases sensitivity to crime-related objects, white-priming has no effect in either direction. This theoretical claim differs from the claims made in Eberhardt et al. (2004) because the hypothetical data are different.

Although it might seem like good science to build a theory solely on observed data, using this approach the conclusions derived from the data tend to fit noise (due to random sampling) in the data as well as any signal. A model or theory that perfectly fits empirical data (in this case at the level of significant or non-significant outcomes) tends to “over fit” the data by proposing a theoretical basis for noise. Such a theory will not do a good job predicting future data because the random noise in a new data set will be different than the noise in the original data set.

Returning to the various outcomes of the white-priming effect; given the reported data, the estimated probability of significance for the *t*-test comparing the white-prime and no-prime conditions (for crime-relevant objects) is around 0.54. This means that due to random sampling, replication experiments of this type will draw different conclusions almost equally often. If one believes that empirical findings should replicate in order to support a theoretical claim, this randomness suggests that researchers should not have much confidence in the theoretical conclusion derived from this test.

## Trying to improve the experiments in Eberhardt et al. (2004)

**Figure 3:**
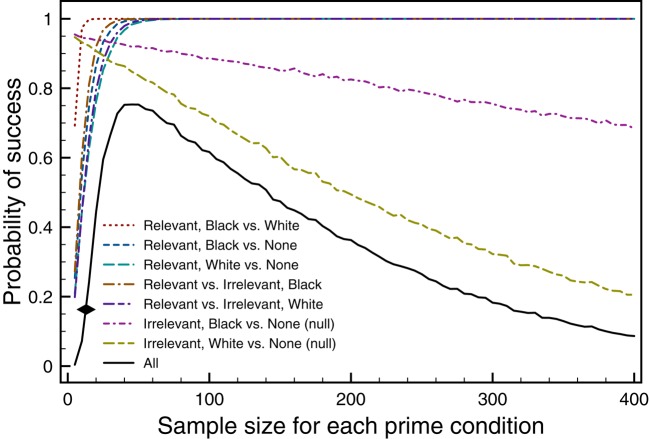
**Each colored line shows the estimated probability of success as a function of sample size for a test from study 1 of Eberhardt et al. (2004)**. The black line shows the estimated success probability for all of the tests. Each point is based on 10,000 simulated experiments.

When doubts are raised about a set of empirical findings, a common approach in psychological science is to run a new experiment. It might seem that a good way to test the theoretical claims in study 1 of Eberhardt et al. (2004) would be to run the same experiment with a larger sample size. Indeed, when empirical support for a theoretical claim is based on a significant hypothesis test, larger samples increase the **power** of the test, so that the study is more likely to find an effect if it exists. However, when the theoretical claims are based on both significant and non-significant tests, there may be limits to the maximum probability of success. These limits for study 1 were investigated with simulated experiments that used the means, standard deviations, and correlations reported by Eberhardt et al. (2004) as population values and varied the sample size for each prime condition. (R source code for the simulations is in the Supplemental Material). The colored lines in Figure 3 plot the estimated probability of success for each of the seven tests used by Eberhardt et al. (2004) as a function of sample size (assuming the same sample size for each priming condition). For the five tests where success corresponds to producing a significant result, the probability of success increases with sample size and converges on the maximum value of 1 at around a sample size of 50. For the two tests where success corresponds to producing a non-significant result, the probability of success decreases with sample size (because some random samples show significant differences).

KEY CONCEPT 5. Power: In a hypothesis test, power is the probability of picking a random sample that produces a statistically significant outcome. The power calculation requires specification of an effect size, experimental design, and sample size(s).

The black line in Figure 3 indicates the probability of all seven tests being successful (significant or non-significant as needed), and the black diamond indicates the average sample size used by Eberhardt et al. (2004). For this line, as sample size increases to 45 participants per condition the estimated success probability increases to a maximum value of 0.753. For sample sizes larger than 45 the estimated probability of all the tests being successful decreases because the tests that should produce non-significant outcomes start to produce significant outcomes. The net result is that, based on the knowledge we have about the means, standard deviations, and correlations from the original experiments, large sample experiments will be unlikely to successfully replicate the full set of findings.

Three of the other experiments in Eberhardt et al. (2004) used only significant outcomes as the basis of their theoretical claims, so larger samples should only increase the probability of replication success. In the large sample limit, the success probability should be 1.0, if the effects are real. However, the claims from study 5 in Eberhardt et al. (2004) depended on both significant and non-significant outcomes from the hypothesis tests. Similar to Figure 3, simulated experiments suggest that the maximum possible success probability for study 5 is 0.465, which occurs for 45 participants in each of four conditions.

Thus, based on the statistics reported by Eberhardt et al. (2004), if a scientist attempts to replicate all five experiments, the best chance of getting the same pattern (significance and non-significance) of results as the original studies is to use very large sample sizes for studies 2–3 (to give a power close to 1.0) and sample sizes of 45 for every condition in studies 1 and 5. The resulting estimated probability of success across all 5 studies would be 0.753 × 1.0 × 1.0 × 1.0 × 0.465 = 0.348, which means that even with optimal sample sizes, there is approximately a one in three chance that a replication of all the studies in Eberhardt et al. (2004) would be fully successful. This seems like rather low odds to motivate a set of replication studies that need several hundred participants.

It may be that the means, standard deviations, and correlations reported by Eberhardt et al. (2004) are not similar to the population values. If so, then researchers need other information (perhaps from other studies or from other theories) to identify sample sizes that would be a good test of the theoretical claims in Eberhardt et al. (2004). An even better approach might be to design entirely new types of studies to investigate these issues. Direct replication is not always an appropriate method to test scientific ideas (see also Rotello et al., 2015). A fruitful approach is to design experiments to address different criteria than standard hypothesis testing approaches. For example, Bayesian analysis methods can identify evidential support for the null hypothesis (e.g., Rouder et al., 2009), so increasing sample sizes inevitably leads to evidential clarity.

## Conclusions

Several commentaries on previous TES analyses have suggested that the TES analysis is unfair (Galak and Mayvis, 2012; Elliot and Maier, 2013; Spellman, 2015), does not answer a relevant question (Simonsohn, 2012, 2013; Morey, 2013; Fabrigar and Wegener, 2016), or has been misapplied (Balcetis and Dunning, 2012; Johnson, 2013; Dias and Ressler, 2014; van Boxtel and Koch, 2016). The concerns about fairness largely reflect misinterpreting a scientific critique as a personal attack (Francis, 2013b,c, 2016a). It is true that some problems identified by a TES analysis may reflect editorial decisions rather than the wishes of the authors, but identifying responsibility for such decisions is a distinct issue that is not resolved by a TES analysis. Concerns about whether the TES answers a relevant question reflect improperly drawn inferences from an analysis of a single article to the general field and misunderstandings about the interpretation of the TES analysis (Francis, 2012b, 2013a,b, in press), especially regarding the relation between reported data and theoretical claims. Concerns about possible misapplications of the TES involve the selection of hypothesis tests that are used to determine success. As discussed above, such selection is based on the reported claims of the original authors, so it is fairly easy to check whether the TES has been applied properly in this regard (Francis, 2012c, 2013a,b, 2016b).

It can be unpleasant to critique articles that are the result of substantial effort by scientists who presumably made a good faith effort to scientifically investigate an important topic. Nevertheless, such critiques are necessary for important topics such as racial bias because good scientific investigations would add valuable insight that could be used in policy decisions and training programs. In contrast, poor scientific investigations, however well intentioned, have the potential to cause true harm.

The problems with the findings in Eberhardt et al. (2004) are hardly unique to psychology (e.g., Ioannidis and Trikalinos, 2007; Francis, 2014b), but some psychologists are actively improving scientific practice. Such efforts include the Many Labs Project (Klein et al., 2014), which explores variability in replication studies across different laboratories; the Reproducibility Project (Open Science Collaboration, 2015), which empirically examined replication success across a set of important findings; promotion and development of software for Bayesian data analysis methods (Rouder et al., 2009; Kruschke, 2010); encouraging data-sharing to enable re-analysis (Nosek et al., 2012); promoting pre-registration of experimental designs and planned analyses (Jonas and Cesario, 2015); and utilizing meta-analytic methods to combine data from multiple underpowered studies (Simonsohn et al., 2014; van Assen et al., 2015). None of these approaches solve all of the problems in the field because science is an inherently difficult endeavor; but keeping these approaches in mind is an important part of improving scientific practice. Ultimately, good science derives from deep understanding, clever design, careful measurement and analysis, and full honesty about the findings and their limitations.

## Author contributions

GF performed all analyses and wrote the text.

### Conflict of interest statement

The author declares that the research was conducted in the absence of any commercial or financial relationships that could be construed as a potential conflict of interest.
